# Estimated energy and nutrient composition of different sources of food waste and their potential for use in sustainable swine feeding programs

**DOI:** 10.1093/tas/txy099

**Published:** 2018-09-07

**Authors:** Leonard Fung, Pedro E Urriola, Lawrence Baker, Gerald C Shurson

**Affiliations:** 1 Department of Animal Science, University of Minnesota, St. Paul, MN; 2 Department of Bioproducts and Biosystems Engineering, University of Minnesota, St. Paul, MN

**Keywords:** amino acids, energy, food waste, nutrients, phosphorus, swine

## Abstract

About 40% of the total food produced in the United States is wasted throughout the supply chain. The objective of this study was to determine the energy and nutrient content and variability of food waste sources generated at different stages within the food supply chain in the Minneapolis–St. Paul, MN, metropolitan area, and their potential for use in swine diets. A total of four waste sources were selected: supermarket (**SM**; retail to consumer), university residential dining hall (**RH**; consumer to postconsumer), a city waste transfer station (**TS**; postconsumer to municipal waste disposal), and household source-separated organic recycling program (**SSO**; postconsumer to municipal waste). Samples were collected (SM: *n* = 22; RH: *n* = 60; TS: *n* = 27; SSO: *n* =12) and analyzed for GE, proximate analyses, minerals, amino acids, and fatty acid concentrations along with lipid peroxidation indicators including peroxide value (**PV**) and thiobarbituric reactive substances (**TBARS**). Data were analyzed using a general linear model that included food waste source as the main factor, and least squared means with adjustment were used for multiple comparisons. Samples of SM food waste contained the greatest (*P* < 0.05) concentration of GE (5,909 kcal/kg) compared with RH, TS, and SSO sources. Calculated NE of SM (3,740 kcal/kg) was also the greatest compared with the three other food waste sources. Food waste from SM, RH, and SSO, but not TS, had greater (*P* < 0.05) calculated NE than published values for corn and soybean meal. Concentrations of Lys (1.82%), Met (0.53%), Thr (1.07%), and Trp (0.27) content were greater in SM than in RH, TS, and SSO, but these concentrations were less than published values for soybean meal. There were no differences (*P* > 0.05) in the phosphorus content of samples among food waste sources (0.30% to 0.64%). PV and TBARS were greatest (*P* < 0.05) in the SSO samples (PV = 82.4 meq/kg oil; TBARS = 2.44 mg malondialdehyde (MDA) eq/g oil) compared with the other three food waste sources. Although the concentrations of nutrients and calculated energy values of the food waste sources were moderately high compared with corn and soybean meal, their composition was more variable (i.e., greater SD of means). Food waste generated upstream (SM) in the food supply chain appears to have greater nutritional value than postconsumer food waste (RH, TS, and SSO), but all sources appear suitable for use in commercial swine diets provided that ME, NE, and nutrient digestibility values are well characterized.

## INTRODUCTION

Food price increases in recent decades have led to discussion on the need to increase agricultural productivity and reduce food waste to ensure food security (von Braun et al., 2008). However, most of the focus has involved developing and implementing new technologies to increase agricultural productivity, with much less attention being devoted to managing the 40% of the food waste generated annually in the United States (Gunders, 2012). Since 1974, food waste in the United States has increased by approximately 50%, and is responsible for 25% of fresh water and 300 million barrels of crude oil consumption annually, due to the inefficiency of diverting food away from its intended use for human consumption and toward its disposal as food waste (Hall et al., 2009). Not only does this amount of food waste lead to significant economic losses, it also causes significant negative social and environmental impacts due to inefficient use of natural resources and the production of greenhouse gases, such as carbon dioxide and methane from landfills that are extensively used for food waste disposal (Adhikari et al., 2006; Hall et al., 2009). Therefore, alternative methods to divert food waste into higher value uses are needed to minimize their environmental impact and promote long-term sustainability of our food system (Dorward, 2012).

Generation of food waste occurs at multiple stages of the food supply chain beginning at production, followed by transportation, handling, storage, processing, packaging, distribution, marketing, consumption, and postconsumption (Parfitt et al., 2010; Lipinski et al., 2013). Within each of these stages, several major food waste generation sources include processing facilities, restaurants, schools, public institutions, grocery stores, and households (Gustavsson et al., 2011).

Limited information has been published previously regarding the chemical characteristics and nutritional value of various sources of food waste and their potential use in animal feeds, and only a few studies have previously investigated the growth performance of pigs fed specific food waste sources (Westendorf et al., 1998; Myer et al., 1999). Therefore, the objective of this study was to characterize the nutritional composition of major food waste sources in the Minneapolis–St. Paul, MN, metropolitan area and their potential for use in swine feeding programs. We hypothesized that food waste generated upstream in the food supply chain provides greater nutritional value due to less contamination with other waste materials (e.g., paper and plastic) than downstream food waste sources.

## MATERIALS AND METHODS

### Food Waste Sources

Four food waste sources representing different food waste generation segments in the food supply chain were identified in the Minneapolis–St. Paul, MN, metropolitan area including retail to consumer (i.e., supermarket; **SM**) waste; consumer to postconsumer food waste (i.e., university residential dining hall; **RH**), postconsumer source-separated organic (**SSO**) waste (i.e., household food waste); and municipal transfer station (**TS**) waste. Specifically, these sources of food waste included the University of Minnesota Saint Paul Campus Residential Hall Dining Services (Falcon Heights, MN), the Hennepin County Recycling Center and Transfer Station (Brooklyn Park, MN), Lunds and Byerlys supermarket (Roseville, MN), and the Hennepin County Organics Recycling Program for Residents (Minneapolis, MN). The RH source provides meals to over 500 students, and the food waste generated at this site is routinely collected and delivered to commercial facilities for composting. The TS source is a city waste collection and TS that is the only facility in the area that accepts organic waste from households and businesses, and includes a wide range of organic materials (food scraps, nonfood organics such as nonrecyclable paper, and biodegradable products) for subsequent composting. The SM source represents a major grocery chain with 37 different stores that sell a variety of food products including bakery, dairy, meat, fruits and vegetables, and restaurant-prepared foods. Finally, the SSO source is a voluntary organic recycling program conducted by the county government and provides both curbside pickup and multiple drop-off locations for residents within Hennepin County. Materials being recycled include food scraps and nonfood organics including food-soiled paper products and other compostable items (e.g., yard waste).

### Food Waste Sample Collection

#### Retail to consumer level—SM.

Food waste samples generated from five departments of the store and the in-store restaurant were collected daily by employees and stored in multiple 120-L recycling bins separated and identified by their respective origins (e.g., dairy, fruits and vegetables, meat, bakery, and restaurant). Subsamples of food waste from each department were obtained directly from the recycling bins using a 8.1 × 10.4 × 34.5 cm^3^ plastic ladle. Each sampling location within the bins was selected randomly regardless of the materials in the bin, and samples collected were placed in a 34.3 × 24.4 × 7 cm^3^ aluminum pan for subsequent drying. Each pan was filled with two full scoops (approximately 400 mL) of each type of food waste, and 10 pans were filled at each collection time from five bins including one fruit and vegetable bin, two meat bins (chicken and beef), one bakery product bin, and one bin from the restaurant. A total of 30 samples were collected from three visits in April, 2015.

#### Consumer to postconsumer level—university residence hall.

Food waste was collected by the dining service employees on a daily basis, stored in 120-L recycling bins, and consisted of discarded food from all three meals from the previous day. Subsampling was carried out by using an 8.1 × 10.4 × 34.5 cm^3^ plastic ladle to remove two full scoops (about 400 mL) of food waste directly from recycle bins. Sampling location within the bins was selected randomly regardless of materials in the bin, and the samples were then placed in 34.3 × 24.4 × 7 cm^3^ aluminum pans for later drying. Each pan was filled with two full scoops (approximately 400 mL) of food waste, and 5 to 10 pans were collected at each sampling time depending on the total volume of the food waste stored in the bins at the time of collection. This resulted in a total of six collections over a 3-mo period from February 2015 to April 2015.

#### Consumer to postconsumer level—household SSO waste recycling program.

Three of seven drop-off SSO locations in the program were randomly selected for collection, which included Audubon Park, Pearl Park, and Armatage Park (Minneapolis, MN). Organic waste generated from households in these communities was collected by residents in program-specific recycling bags (Biodegradable Products Institute, NY) and delivered to these three locations. Samples were directly collected from individual drop-off containers with 1 bag/container (12 L/bag). A total of 48 samples were collected over 2-wk periods, which involved six visits at two different times (August 2015 and January 2016). Samples collected on the same day from each location were pooled to form one representative sample from each location and day of collection.

#### Postconsumer to municipal organic waste level—organic waste TS.

Organic waste at the TS was piled in a 4 × 4 m^2^ bunker to be later transferred by trucks to a composting facility. Samples were collected by dividing the area into 9 quadrants in which 1 quadrant was approximately 1.7 m^2^, and organic waste from each quadrant was subsampled using a shovel. Samples from each quadrant were randomly selected and placed into two 34.3 × 24.4 × 7 cm^3^ aluminum pans. Nine pans of waste (approximately 400 mL/pan) were collected during each of three visits over a 2-mo period from February 2015 to March 2015.

### Processing of Samples

After each collection, samples in trays were weighed using a Tanita 144 laboratory scale (Arlington Heights, IL), and weights were recorded after subtracting the aluminum tray weight from the total weight. Next, samples were dried in a forced-air oven at 60 °C for 72 h. After the 72-h period, samples were removed from the oven and weighed using the same scale to determine the dry weight of each sample. Subsequently, each tray of samples was ground and mixed individually using a robot coupe Blixer 3 Series D 3 ½ Qt (Ridgeland, MS) into a fine power. One hundred grams of powder was subsampled from each collection tray and sent to a commercial laboratory for chemical analysis.

### Chemical Analysis

All samples were submitted to Minnesota Valley Testing Laboratory (New Ulm, MN) for proximate analysis. Chemical analysis was conducted using Association of Official Analytical Chemists (2012) methods for CP (Method 990.03), ether extract (**EE**; Method 920.39), ash (Method 942.05), calcium (Method 985.01), phosphorus (Method 985.01), sodium (Method 985.01), NDF (Method 2002.04), and ADF (Method 973.18). Starch was measured using an enzymatic extraction and glucose measurement method that was developed by Minnesota Valley Testing Laboratories. GE was determined by using an adiabatic oxygen bomb calorimeter (Parr Instrument Co., Moline, IL). Samples were also submitted to University of Missouri Experiment Station Chemical Laboratories (Columbia, MO) for analysis using AOAC (2006) procedures for fatty acid profile (Method 996.06), amino acid profile (Method 982.30), peroxide value (**PV**) (Method 965.33). Thiobarbituric acid reactive substances (**TBARS**) content was determined according to the current protocols in Analytic Chemistry (2001; D2. 4.1—D2.4.18).

### Energy and Iodine Value Calculations

#### Energy calculations.

Three published equations from the “Nutrients Requirements of Swine” (NRC, 2012) were used to estimate the DE, ME, and NE content of the food waste from chemical composition using analyzed chemical composition:

$$\begin{array}{l} \text{DE},\text{ kcal}/\text{kg DM }=\text{ 1},\text{161 }+\;\left(0.\text{749 }\times \text{ GE},\text{ kcal}/\text{kg DM}\right)\;-\;\left(\text{4}.\text{3 }\times \text{ Ash},\text{ g}/\text{kg DM}\right)\;-\\ \;\left(\text{4}.\text{1 }\times \text{ NDF},\text{ g}/\text{kg DM}\right) \end{array}$$

(Noblet and Perez 1993)

$$\begin{array}{l} \text{ME},\text{ kcal}/\text{kg DM }=\text{ 4},\text{194 }-\;\left(\text{9}.\text{2 }\times \text{ Ash},\text{ g}/\text{kg DM}\right)\;+\;\\ \left(\text{1}.0\;\times \text{ CP},\text{ g}/\text{kg DM}\right)\;+\;\left(\text{4}.\text{1 }\times \text{ EE},\text{ g}/\text{kg DM}\right)\;-\;\left(\text{3}.\text{5 }\times \text{ NDF},\text{ g}/\text{kg DM}\right) \end{array}$$

(Noblet and Perez 1993)

$$\begin{array}{l} \text{NE},\text{ kcal}/\text{kg DM }=\;\left(0.\text{726 }\times \text{ ME},\text{ kcal}/\text{kg DM}\right)\;+\;\left(\text{1}.\text{33 }\times \text{ EE},\text{ g}/\text{kg DM}\right)\;+\;\\ \left(0.\text{39 }\times \text{ Starch},\text{ g}/\text{kg DM}\right)\;-\;\left(0.\text{62 }\times \text{ CP},\text{ g}/\text{kg DM}\right)\;-\;\left(0.\text{83 }\times \text{ ADF},\text{ g}/\text{kg DM}\right) \end{array}$$

(Noblet et al., 1994)

#### Iodine value calculations.

Iodine value (**IV**) equations from NRC (2012) were used to calculate both total IV and iodine value product (**IVP**) based on the fatty acid profiles of the food waste samples:

$$\begin{array}{l} \text{Total IV}=\left(\text{C16}:\text{1 }\times \;0.\text{9976}\right)\;+\;\left(\text{C18}:\text{1 }\times \;0.\text{8985}\right)\;+\;\left(\text{C18}:\text{2 }\times \text{ 1}.\text{8}0\text{99}\right)\;+\;\left(\text{C18}:\text{3 }\times \text{ 2}.\text{7345}\right)\;+\;\\ \left(\text{C2}0:\text{1 }\times \;0.\text{8173}\right)\;+\;\left(\text{C2}0:\text{4 }\times \text{ 3}.\text{3343}\right)\;+\;\left(\text{C2}0:\text{5 }\times \text{ 4}.\text{1956}\right)\;+\;\\ \left(\text{C22}:\text{1 }\times \;0.\text{7496}\right)\;+\;\left(\text{C22}:\text{5 }\times \text{ 3}.\text{8395}\right)\;+\;\left(\text{C22}:\text{6 }\times \text{ 4}.\text{6358}\right) \end{array}$$

and

IVP = (IV of ingredient EE) × (% EE in the ingredient) × 0.1

### Statistical Analysis

Individual samples from each location and collection tray was considered as the experimental unit for all analyses. Chemical composition data, calculated energy and IVs, and lipid peroxidation data were analyzed using the GLM procedure of SAS 9.3 (SAS Inst. Inc., Cary, NC). Food waste source was considered as a fixed effect. Data were analyzed using a general linear model that included food waste source as the main factor, and least squared means with adjustment were used for multiple comparisons. Significant differences were designated if *P* ≤ 0.05 and trends were noted when 0.05 < *P* < 0.10.

## RESULTS AND DISCUSSION

### Analyzed Chemical Composition and Calculated Energy Values of Food Waste Sources

All analyzed nutrient values are expressed on a DM basis. No differences were observed in the moisture content among food waste sources during the initial drying process (60 °C for 72 h), and subsequent DM analysis after the initial drying. However, the initial moisture content of all the food waste sources was greater than 60% (Table 1). Because dry feeding systems are the predominant form used in the U.S. pork industry (Richert and DeRouchey, 2010), the high moisture content of all sources of food waste requires drying before they can be incorporated into diets in commercial feed mills used in pork production systems. The initial drying process of 60 °C for 72 h was effective in reducing the moisture content to 5% to 10%, which is common for feed ingredients such as corn (11.7%) and soybean meal (4.4%). High moisture content in food waste can increase the susceptibility to microbial growth and spoilage, and as a result, requires thermal heating to remove moisture before it can be fed to swine (Kabak et al., 2006).

**Table 1.** Analyzed nutrient composition and calculated energy content (DM basis) of food waste from preconsumer (SM), postconsumer (university dining hall and household SSOs), and municipal organic waste collection (TS) facilities in Minnesota compared with nutritional composition of corn and dehulled, solvent-extracted soybean meal (NRC, 2012)

	SM (*n* = 22)		University dining hall (*n* = 60)		TS (*n* = 27)		SSOs (*n* = 12)			Soybean meal^1^		Corn^2^	
Item	Mean	SD	Mean	SD	Mean	SD	Mean	SD	SEM	Mean	SD	Mean	SD
Moisture 1^3^, %	61.08	17.16	60.85	12.03	69.07	10.44	72.52	10.30	2.42	NA	NA	NA	NA
Moisture 2^4^, %	8.33	12.49	7.02	4.76	9.84	6.22	5.15	0.69	1.46	10.02	2.62	11.69	2.41
Crude protein, %	25.51^a^	13.50	18.90^b^	3.15	17.71^b^	6.86	13.53^b^	3.73	1.49	53.05	2.30	9.33	0.93
EE, %	31.57^a^	18.96	13.58^b^	3.37	11.09^b^	6.35	10.60^b^	5.51	2.05	1.69	0.91	3.94	0.78
Ash, %	7.73^a^	4.59	5.01^b^	1.27	7.73^a^	3.60	5.58^a,b^	2.22	0.55	7.47	0.51	1.47	0.32
Ca, %	0.98^a^	1.04	0.25^b^	0.21	1.02^a^	0.94	0.85^a^	0.89	0.14	0.37	0.10	0.02	0.01
P, %	0.64	0.60	0.30	0.07	0.46	0.36	0.31	0.16	0.11	0.79	0.09	0.29	0.05
Na, %	0.77^a^	0.38	0.85^a^	0.17	0.72^a^	0.87	0.29^b^	0.10	0.08	0.09	0.05	0.02	0.00
NDF, %	12.37^b^	8.05	6.72^b^	9.14	22.99^a^	12.53	24.59^a^	6.80	2.16	9.12	2.90	10.32	1.97
ADF, %	12.92^b^	8.27	5.29^c^	6.88	19.76^a^	10.45	17.44^ab^	4.87	1.69	5.87	2.43	3.26	0.83
Starch, %	11.57^b^	13.45	42.11	10.57	16.28^b^	9.62	12.50^b^	5.53	2.36	2.10	NA	70.83	4.61
Energy (kcal/kg)													
GE^5^	5,909^a^	1,016	5,419^a^	349	4,829^b^	486	4,455^b^	309	140	4,730	192	3,933	86
DE^6^	5,016^a^	1,152	4,418^b^	425	3,421^c^	721	4,552^b^	283	120	4,022	184	3,451	111
ME^7^	4,832^a^	1,274	4,188^b^	422	3,198^c^	575	4,114^b^	44	120	3,660	NA	3,395	NA
NE^8^	3,740^a^	1,188	3,221^b^	407	2,252^c^	532	2,983^b^	40	110	2,319	NA	2,672	NA

NA = not applicable.

^a,b,c^Means with different superscripts within a row differ (*P* < 0.05).

^1^Soybean meal, dehulled solvent extracted mean and standard deviation values were obtained from NRC (2012).

^2^Yellow dent corn mean and standard deviation values were obtained from NRC (2012).

^3^Moisture content after initial drying process at 60 °C for 72 h.

^4^Moisture content of initially dried samples at 135 °C for 2 h.

^5^GE (kcal/kg) determined by adiabatic bomb calorimetry.

^6^Calculated DE (kcal/kg) = 1,161 + (0.749 × GE, kcal/kg) − (4.3 × Ash, g/kg) − (4.1 × NDF, g/kg) (Noblet and Perez 1993).

^7^Calculated ME (kcal/kg) = 4,194 − (9.2 × Ash, g/kg) + (1.0 × CP, g/kg) + (4.1 × EE, g/kg) − (3.5 × NDF, g/kg) (Noblet and Perez 1993).

^8^Calculated NE (kcal/kg) = (0.726 × ME, kcal/kg) + (1.33 × EE, g/kg) + (0.39 × Starch, g/kg) − (0.62 × CP, g/kg) − (0.83 × ADF, g/kg) (Noblet et al. 1994).

SM food waste had the greatest concentration of CP (25.5%) and EE (31.6%) compared with all the other sources (*P* < 0.05). The relatively high concentration of CP and EE in the SM samples was a result of the high proportion of meat products in the collected waste. There were no differences in CP and EE content among RH (18.90% CP, 13.58% EE), TS (17.71% CP, 11.09% EE), and SSO (13.53% CP, 10.60% EE) sources. The relatively high CP and EE content of these food waste sources suggest that they may be valuable feed ingredients in swine diets because energy provided by EE and protein are the two most expensive nutritional components (Kerr et al., 2015; Zhou et al., 2015). When comparing the CP and EE content in the food waste sources with that in corn and soybean meal, CP content (26% to 14%) was intermediate between corn (9.3%) and dehulled, solvent-extracted soybean meal (47.2%), whereas EE content in food waste (31.6% to 10.6%) exceeded that in corn (3.9%) and soybean meal (1.7%).

The fiber content of TS (23.0% NDF, 19.8% ADF) and SSO (24.6% NDF, 17.4% ADF) was greater (*P* < 0.05) for SM (12.4% NDF, 12.9% ADF) and RH (6.7% NDF, 5.3% ADF) samples. The high proportion of fiber in both TS and SSO samples was expected because these food waste sources were composed primarily of fruit and vegetable waste. Vegetables and fruit waste contain a significant amount fiber on a DM basis, and as a result, would be expected to reduce ME and NE content of these food waste sources for swine. The inclusion of high fiber ingredients in swine diets has been shown to reduce energy and nutrient digestibility, increase digesta passage rate, and reduce efficiency of growth (Kennelly and Aherne, 1980; Myrie et al., 2008; Kerr and Shurson, 2013; Pérez de Nanclares et al., 2017). However, mechanical processing (e.g., pelleting and micronizing) and the addition of exogenous enzymes have been shown to increase the utilization of nonstarch polysaccharides in some high fiber ingredients (Kerr and Shurson, 2013).

Starch content was greater (*P* < 0.05) in the RH samples (42.1%) compared with SM (11.6%), TS (16.3%), and SSO (12.5%) sources, and the relatively high starch content in the RH samples was likely due to the high proportion of bakery goods and pizza waste. Starch is a highly DE source in animal feed (Keys and DeBarthe, 1974; Noblet, 2000), and provides high energy and economic value in swine diets.

SM (7.7%) and TS (7.7%) samples had a greater (*P* < 0.05) concentration of ash compared with RH (5.0%) and SSO (5.6%) samples. Calcium content was greater (*P* < 0.05) in SM (0.98%), TS (1.02%), and SSO (0.85%) samples compared with RH samples (0.25%). However, sodium content was greater (*P* < 0.05) in the SM (0.77%), RH (0.85%), and TS (0.72%) samples compared to SSO (0.29%) samples. However, there were no differences in phosphorus content among these four food waste sources. The relatively high phosphorus and calcium content in the SM waste was likely a result of the high proportion of meat, dairy, and processed deli products in this food waste mixture. Yet, the concentrations of phosphorus and calcium in all of these food waste sources (< 1%) are not great enough to be considered as major sources of these minerals in swine diets.

Energy is the most expensive nutritional component in swine diets. Therefore, it is very important to estimate the ME or NE content of feed ingredients before feed formulation (Kerr et al., 2015). The analyzed GE content was greater (*P* < 0.05) for the SM samples (5,909 kcal/kg) and RH samples (5,419 kcal/kg) compared with TS (4,829 kcal/kg) and SSO (4,455 kcal/kg) samples. Food waste from SM had greater (*P* < 0.05) calculated DE (5,016 kcal/kg), ME (4,832 kcal/kg), and NE (3,740 kcal/kg) compared with the other food waste sources, whereas TS samples had the least (*P* < 0.05) DE (3,421 kcal/kg), ME (3,198 kcal/kg), and NE (2,252 kcal/kg). The DE, ME, and NE content of RH and SSO samples were similar despite the differences in GE content. When compared to corn (3,933 kcal/kg GE; 3,451 kcal/kg DE; 3,395 kcal/kg ME; and 2,672 kcal/kg NE), which is the major energy contributor in U.S. swine diets, SM, RH, and SSO samples had greater GE, calculated DE, ME, and NE content, whereas TS samples had less estimated DE, ME, and NE content than corn due to its high concentration of NDF. Thus, adding food waste sources from SM, RH, and SSO to swine diets would provide greater ME and NE than corn. However, the accuracy of the energy prediction equations used in this study have not been validated for use in food waste sources. Therefore, the DE, ME, and NE content of food waste sources should be determined experimentally to verify the accuracy of their energy content before feeding them to swine (Kil et al., 2013).

### Amino Acid Profile of Food Waste Sources

Considering that Lys, Met, Thr, and Trp are the first four limiting amino acids in corn–soybean meal-based diets for swine, SM food waste samples had the greatest (*P* < 0.05) concentration of all these amino acids (1.82% Lys, 0.27% Trp, 1.07% Thr, and 0.53% Met) compared with the other three food waste sources (Table 2). Furthermore, there were no differences in Lys, Met, and Thr content among RH, TS, and SSO sources. In contrast, TS and SSO had less (*P* < 0.05) Trp content (0.13% and 0.08%, respectively) compared with samples of SM (0.27%) and RH (0.20%). Amino acid content, digestibility, and their proportions relative to the first limiting are important factors when formulating diets to optimize growth performance and lean tissue protein accretion in pigs (Wang and Fuller, 1989; Kerr and Easter, 1995; Stein et al., 2007). The Lys, Trp, Thr, and Met content in all food waste sources was greater than corn (0.28% Lys, 0.07% Trp, 0.32% Thr, and 0.20% Met), but less than soybean meal (3.29% Lys, 0.73% Trp, 2.07% Thr, and 0.73% Met). Swine diets should be formulated on a standardized ileal digestible amino acid basis rather than total amino acid basis to accurately supply amino acids without excess or deficiencies (Sauer and Ozimek, 1986). Because of differences in chemical characteristics, and the extent of previous processing and heating of the food waste sources evaluated in this study, the digestibility of amino acids is uncertain (Sauer et al., 1991; Stein et al., 2007). Therefore, direct in vivo determination of the digestibility of amino acids is necessary before formulating diets containing these food waste sources (Stein et al., 2007).

**Table 2.** Indispensable amino acid content (DM basis) of food waste from preconsumer (SM), postconsumer (university dining hall and household SSOs), and municipal organic waste collection (TS) facilities in Minnesota compared with corn and dehulled, solvent-extracted soybean meal (NRC, 2012)

	SM (*n* = 17)		University dining hall (*n* = 55)		TS (*n* = 22)		SSOs (*n* = 12)			Soybean meal^1^		Corn^2^	
Item	Mean	SD	Mean	SD	Mean	SD	Mean	SD	SEM	Mean	SD	Mean	SD
Arginine	1.63^a^	1.09	0.79^b^	0.19	0.59^b^	0.27	0.58^b^	0.20	0.10	3.83	0.26	0.42	0.05
Histidine	0.72^a^	0.42	0.44^b^	0.12	0.29^c^	0.14	0.33^bc^	0.09	0.04	1.42	0.10	0.27	0.05
Isoleucine	1.08^a^	0.56	0.72^b^	0.16	0.54^b^	0.24	0.52^b^	0.13	0.06	2.38	0.18	0.32	0.06
Leucine	1.96^a^	1.03	1.27^b^	0.29	0.96^b^	0.41	0.94^b^	0.22	0.11	4.02	0.27	1.09	0.15
Lysine	1.82^a^	1.27	0.77^b^	0.32	0.67^b^	0.31	0.63^b^	0.22	0.13	3.29	0.19	0.28	0.04
Methionine	0.53^a^	0.34	0.31^b^	0.08	0.22^b^	0.12	0.22^b^	0.07	0.04	0.73	0.08	0.20	0.03
Phenylalanine	1.06^a^	0.51	0.80^b^	0.16	0.58^c^	0.24	0.57^c^	0.12	0.06	2.67	0.19	0.44	0.05
Threonine	1.07^a^	0.62	0.59^b^	0.14	0.47^b^	0.20	0.43^b^	0.12	0.06	2.07	0.11	0.32	0.04
Tryptophan	0.27^a^	0.14	0.20^b^	0.05	0.13^c^	0.06	0.08^c^	0.03	0.02	0.73	0.08	0.07	0.01
Valine	1.23^a^	0.63	0.79^b^	0.18	0.59^b^	0.26	0.66^b^	0.14	0.07	2.48	0.19	0.43	0.05

^a,b,c^Means with different superscripts within a row differ (*P* < 0.05).

^1^Soybean meal, dehulled solvent extracted mean and standard deviation values were obtained from NRC (2012).

^2^Yellow dent corn mean and standard deviation values were obtained from NRC (2012).

Although the concentration of biogenic amines was not determined in food waste sources evaluated in this study, the concentrations of putrescine, cadaverine, spermidine, and spermine should also be determined before adding to swine diets. Biogenic amines are resulting products of the decarboxylation of free amino acids by bacteria found in animal tissues and plants (ten Brink et al., 1990; Salazar et al., 2000). High concentrations of biogenic amines may indicate significant spoilage and degradation of high protein feed ingredients, and feeding diets containing high concentrations of these compounds can result in toxicity and reduction of growth performance in animals (Smith, 1990; Salazar et al., 2000; Teti et al., 2002).

### Fatty Acid Profile and Lipid Quality of Food Waste Sources

The concentration of linoleic acid (C18:2) was greater (*P* < 0.05) in the TS samples (32.8%) compared to SM (15.9%) and SSO (23.3%) sources (Table 3). However, the concentration of linoleic acid in RH samples (29.3%) was not different from the three other food waste sources. Linolenic acid (C18:3) content was also greater (*P* < 0.05) in the TS samples (7.1%) compared with the other three sources, but there were no differences in the linolenic acid content among samples of SM (2.2%), RH (3.8%), and SSO (2.4%). Finally, the concentration of arachidonic acid was not different among samples of SM (0.23%), RH (0.20%), and TS (0.24%) food waste. The SSO samples had less (*P* < 0.05) arachidonic acid content compared with TS samples, but arachidonic acid content was not different from SM and RH samples. The concentration of fatty acids and their relative proportions are important in feeding programs because they supply essential fatty acids such as linoleic and linolenic acid (NRC, 2012). However, feeding high amounts of polyunsaturated fatty acids have been shown to reduce pork fat firmness (Wu et al., 2016; Villela et al., 2017). Thus, it is important to determine the fatty acid composition of lipids in various feed ingredients when formulating growing-finishing pig diets to achieve acceptable pork fat quality. It is difficult to achieve the desired balance fatty acids in diets because there are multiple fatty acids present in various concentrations among feed ingredients. Therefore, IVP and other carcass fat quality predictions have been developed to simplify diet formulation. For example, the IV of distillers’ corn oil, soybean oil, and palm (vegetable-derived oils widely available for feeding swine around the world) is relatively high compared with that of palm oil (Lindblom et al., 2017). Also, choice white grease and tallow are commonly used animal fat sources in swine diets, and have an IVP less than distillers corn oil (Davis et al., 2015). Relative to these common supplemental lipid sources, food waste has a concentration of polyunsaturated fatty acids that is comparable to corn oil. However, the fatty acid composition in various food waste sources is dependent on the origin of the fats and oils present in specific food waste sources.

**Table 3.** Fatty acid composition (% of EE), lipid peroxidation indicators, IV, and IVP of food waste (DM basis) from preconsumer (SM), postconsumer (university dining hall and household SSOs), and municipal organic waste collection (TS) facilities in Minnesota compared with corn and dehulled, solvent-extracted soybean meal (NRC, 2012)

	SM (*n* = 17)		University dining hall (*n* = 55)		TS (*n* = 22)		SSOs (*n* = 12)			Soybean meal^1^		Corn^2^	
Measurement	Mean	SD	Mean	SD	Mean	SD	Mean	SD	SEM	Mean	SD	Mean	SD
EE, %	31.57^a^	18.96	13.58^b^	3.37	11.09^b^	6.35	10.60^b^	5.51	2.05	1.52	0.91	3.94	0.78
Linoleic acid (18:2n6), %	15.88^b^	9.38	29.31^ab^	5.48	32.76^a^	11.74	23.34^b^	6.36	1.75	49.17	NA	44.24	NA
Linolenic acid (18:3n3), %	2.19^b^	2.47	3.82^b^	1.12	7.05^a^	6.27	2.43^b^	0.75	0.69	1.52	NA	1.37	NA
Arachidonic acid (20:4n6), %	0.23^ab^	0.12	0.20^ab^	0.11	0.24^a^	0.18	0.13^b^	0.10	0.03	0.00	NA	NA	NA
*Lipid peroxidation*													
PV, meq/kg lipid	66.44^b^	18.17	62.33^b^	15.00	62.16^b^	18.38	82.43^a^	50.88	4.88	NA	NA	NA	NA
TBARS, mg MDA eq/g lipid	0.17^b^	0.05	0.16^b^	0.05	0.18^b^	0.05	2.44^a^	1.55	0.11	NA	NA	NA	NA
*Lipid composition*													
IV^3^	68.15^c^	17.06	86.60^ab^	9.53	90.60^a^	16.29	78.63^b^	9.01	2.77	NA	NA	NA	NA
IVP^4^	211.29^a^	81.02	95.22^b^	25.35	77.84^b^	46.02	71.42^b^	39.34	9.88	NA	NA	NA	NA

NA = not applicable.

^a,b,c^Means with different superscripts within a row differ (*P* < 0.05).

^1^Soybean meal, dehulled solvent extracted mean and standard deviation values were obtained from NRC (2012).

^2^Yellow dent corn mean and standard deviation values were obtained from NRC (2012).

^3^IV = calculated iodine value of extracted lipid = (C16:1 × 0.9976) + (C18:1 × 0.8985) + (C18:2 × 1.8099) + (C18:3 × 2.7345) + (C20:1 × 0.8173) + (C20:4 × 3.3343) + (C20:5 × 4.1956) + (C22:1 × 0.7496) + (C22:5 × 3.8395) + (C22:6 × 4.6358) (NRC, 2012).

^4^IVP = calculated IV product of extracted lipid = (IV of ingredient EE) × (% of EE in the ingredient) × (0.1) (NRC, 2012).

Because of the relatively high EE content in food waste, exposure to high cooking and thermal processing conditions during drying, lipid peroxidation may occur. Therefore, PV and TBARS were evaluated using lipids extracted from these food waste sources. PV was greater (*P* < 0.05) in SSO samples (82.43 meq/kg oil) compared with the other three sources, but there were no differences between SM (66.44 meq/kg oil), RH (62.33 meq/kg oil), or TS (62.16 meq/kg oil) samples. The same pattern was also observed for TBARS values, where SSO samples (2.44 mg MDA eq/g oil) had greater (*P* < 0.05) TBARS values compared with the three other sources, but there were no differences between SM (0.17 mg MDA eq/g oil), RH (0.16 mg MDA eq/g oil) and TS (0.18 mg MDA eq/g oil) sources. A relatively high concentration of TBARS was observed in SSO waste, which was likely due to high temperatures used during cooking in the households, as well as the extended storage time at collection sites with thermal and oxygen exposure, before the sampling took place.

Feeding peroxidized lipids to pigs has been shown to reduce growth performance, impair immune function, and reduce pork quality (Takahashi and Akiba, 1999; Wood et al., 2004; Hanson, 2014; Kerr et al., 2015; Hung et al., 2017). Although DeRouchey et al. (2004) suggested that feeding thermally oxidized lipid with a PV > 40 mEq/kg to nursery pigs can reduce ADG and ADFI, there are no well-established maximum tolerable limits of feeding oxidized lipids to swine because of poor association of the variable concentrations of chemically diverse lipid oxidation products and growth performance in pigs (Shurson et al., 2015). In addition, high concentrations of TBARS in the feed can lead to detectable off flavors in the meat, with the range of 0.5 to 1.0 mg MDA/kg in final meat products (Greene and Cumuze, 1982). However, addition of antioxidants to feed ingredients and complete feeds containing high lipid content may mitigate the adverse effect of feeding peroxided lipid to the animals, even though antioxidants added to feed does not reverse the state of peroxidation (Sherwin and Products, 1978; Kerr et al., 2015). Thus, it is important to minimize exposure to high heat and oxygen when processing the food waste into animal feed, as well as the length of time it is stored before collection and processing. Unfortunately, there are no standards for the maximal tolerable dietary concentrations of lipid oxidation products in swine diets, and the use of single indicators (e.g., PV or TBARS) are insufficient to accurately predict pig growth performance responses (Shurson et al., 2015). Therefore, it is unknown whether the PV and TBARS concentrations determined in this study are of the magnitude to negatively affect growth performance when added to pig diets.

To provide a perspective of the quantity and nutritional value of food waste sources relative to the nutritional requirements of growing pigs, we estimated the per capita food waste generation in the Minneapolis–St. Paul Metropolitan area (population = 3.28 million) using data (corrected to dry weight) provided by the Natural Resources Defense Council (Gunders et al., 2017) and the DE, CP, and P content from our analysis of food waste sources (Table 1). These calculations suggest that the food waste generated in the Minneapolis–St. Paul metropolitan area could potentially provide 694 million Mcal DE, 25.8 million kg protein, and 0.6 million kg P per year. We estimated that a growing pig would require 900 Mcal DE, 60 kg of protein, and 2 kg of P to grow from 25 to 125 kg BW. Therefore, annual food waste generated from this geographic region alone could provide a sufficient amount of DE to feed 770,000 pigs, a sufficient amount of protein to feed 430,000 pigs, and sufficient P to feed 300,000 thousand pigs.

In conclusion, food waste collected from an SM, university residence dining hall, TS, and household SSO sources varied in chemical composition due to the types of food waste and the segment of the food supply chain where they were collected. These results support our hypothesis that food waste from upstream sources (i.e., SM and residence dining hall) are less diluted with other nonfood organics (i.e., TS and household SSOs) and have greater feeding value in swine diets than downstream sources. However, significant variability was observed within each source of food waste, which may require blending of multiple batches within source to provide more consistency in nutrient content to the feed industry. The concentrations of biogenic amines and peroxidized lipids should be considered when evaluating the use and nutritional value of various food waste sources in swine diets. Although food waste sources evaluated in this study appear to be suitable sources of energy and nutrients for commercial swine diets, further research is needed to directly determine ME and NE content, as well as amino acid and phosphorus digestibility when using these food waste sources in precision swine feeding programs.

## FUNDING

Minnesota Discovery, Research and Innovation Economy (MnDRIVE).

*Conflict of interest statement*. None declared.
